# Supplementary material for: Age Matters: A Comparative Study of RF Heating of Epicardial and Endocardial Electronic Devices in Pediatric and Adult Phantoms during Cardiothoracic MRI
Source: Diagnostics (Basel). 2023 Sep 2;13(17):2847. doi: 10.3390/diagnostics13172847 (PMC10486594; doi:10.3390/diagnostics13172847)
Supplement: Supplementary file 1 [file diagnostics-13-02847-s001.zip › diagnostics-2276652-supplementary.pdf]

Supplementary Information Figure S1

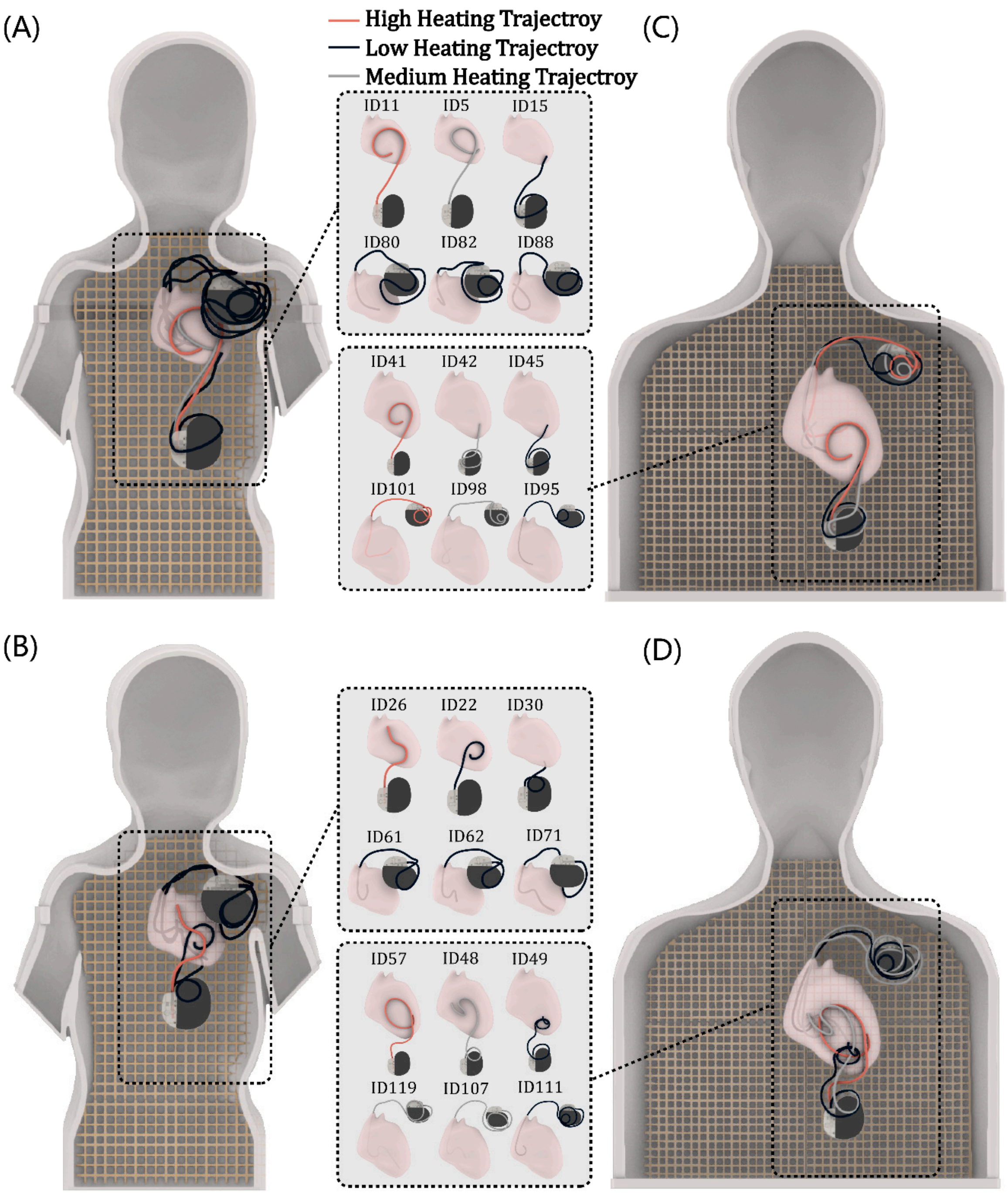

Figure S1: Device configurations used in test-retest experiments. (A) Pediatric phantom with 25-cm epicardial and 45-cm endocardial lead trajectories, (B) Pediatric phantom with 15-cm epicardial and 35-cm endocardial lead trajectories, (C) Adult phantom with 25-cm epicardial and 45-cm endocardial lead trajectories, (D) Adult phantom with 35-cm epicardial and 58-cm endocardial lead trajectories.

Supplementary Information Table S1

Table S1: Temperature rises for test-retest experiments. Device IDs correspond to configurations shown in Figure S1.

Pediatric Phantom	Retest temp[°C]	Original temp[°C]	Adult Phantom	Retest temp[°C]	Original temp[°C]
ID11	10.51	12.34	ID41	13.06	12.06
ID5	4.52	4.04	ID42	1.41	2.18
ID15	0.52	0.31	ID45	0.57	0.28
ID26	5.35	5.51	ID57	5.86	6.18
ID22	1.23	1.73	ID48	1.2	1.98
ID30	0.86	0.37	ID49	0.27	0.19
ID62	2.47	1.74	ID101	5.97	5.90
ID61	1.38	1.25	ID98	4.58	3.22
ID71	0.51	0.84	ID95	2.42	0.8
ID80	0.35	0.44	ID119	2.43	2.57
ID88	0.21	0.39	ID107	1.53	2.05
ID82	0.04	0.09	ID111	0.37	0.27

Incident E-field simulation

Electromagnetic simulations were implemented in ANSYS Electronics Desktop 2020 R2 (ANSYS Inc., Canonsburg, PA). A pediatric body model was created from segmented MRI images of a 29-month-old child, and an adult body model was created from segmented CT images of an adult. Body models were processed to generate a tetrahedral mesh. A 16-rung high-pass birdcage coil model was created and tuned to 63.6 MHz, mimicking a Siemens 1.5 T Aera body coil. Both pediatric and adult body model were positioned inside the coil at the chest imaging landmark. The input power of the coil was adjusted to generate an average $B_1^+ = 5\ \mu T$ on an axial plane passing through the coil's iso-center (Figure S2).

To improve the accuracy of the simulation, the initial mesh was adjusted to ensure that the maximum element size was less than 20 mm for both body models and less than 10 mm for the birdcage coil. ANSYS Electronics Desktop follows an adaptive mesh scheme with the successive refinement of the initial mesh between iterative passes. Scattering parameters (S-parameters) are evaluated at each port and compared to the previous pass at each adaptive pass. Simulations were considered to be converged when the magnitude of the change in S-parameters between the two consecutive passes fell below a set threshold of 0.001. All simulations converged within five adaptive passes. Mesh characteristics are given in Supporting Information Table S2. Total simulation time was approximately 1 hour for simulation of the pediatric model and 2 hours for the adult model on a DELL server with 1.5 TB memory and 2_Xenon(R) Gold 6140 CPUs, each having 32 processing cores.

Supplementary Information Figure S2

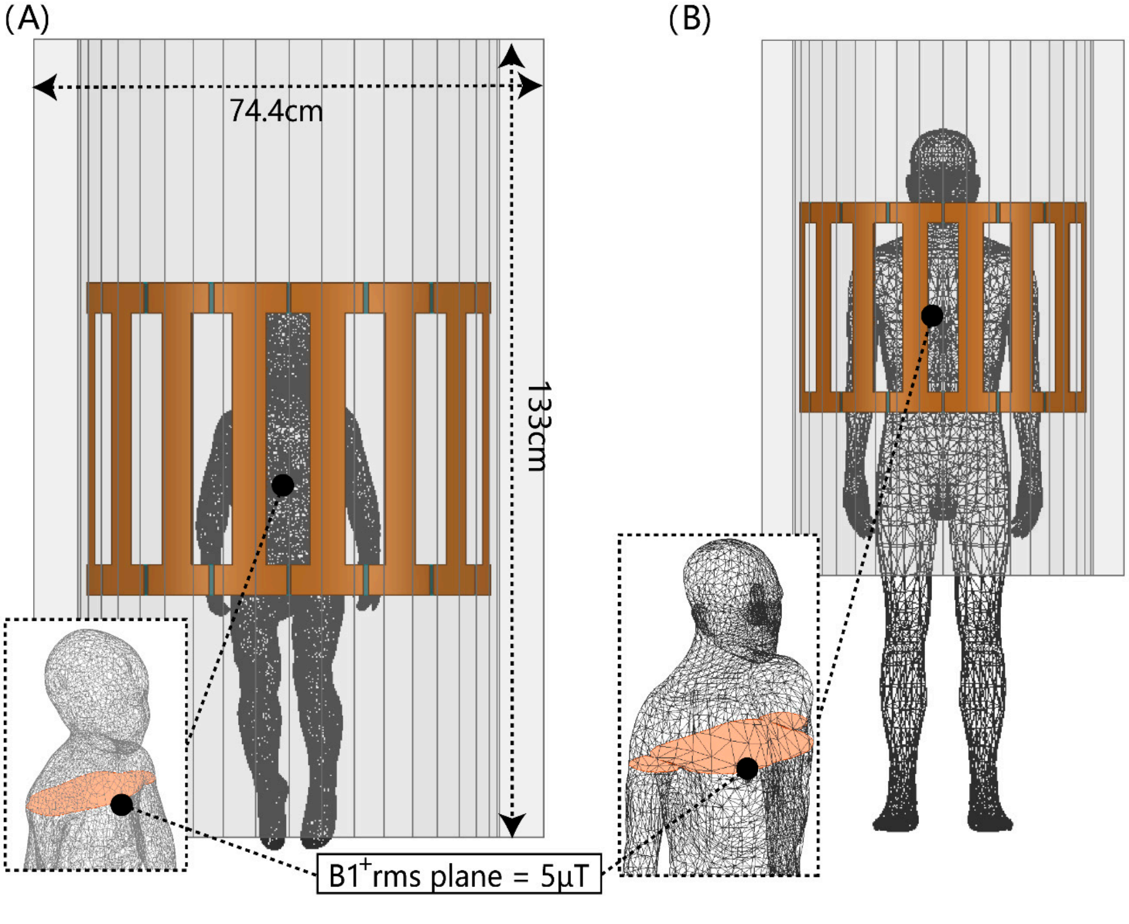

Figure S2: Simulation setup. (A) The homogenous pediatric body model (σ = 0.47 S/m; ε_r = 80) placed inside a model of Siemens 1.5 T Aera body coil with the chest at the iso-center. (B) The homogenous adult body model placed inside a model of Siemens 1.5 T Aera body coil with the chest at the iso-center.

Supplementary Information Table S2

Table S2: Mesh characteristics for the simulations of pediatric and adult body models.

Parts	Num Tets	Min edge length (mm)	Max edge length (mm)	RMS edge length (mm)	Min tet vol (mm^3)	Max tet vol (mm^3)	Mean tet vol (mm^3)	Std Devn (vol) (mm^3)
Child Body	105328	0.46	26.22	14.88	9.72E-05	759.15	126.50	89.70
Adult Body	327401	2.36	22.89	16.04	1.83E-02	704.40	204.46	83.82

Supplementary Information Figure S3

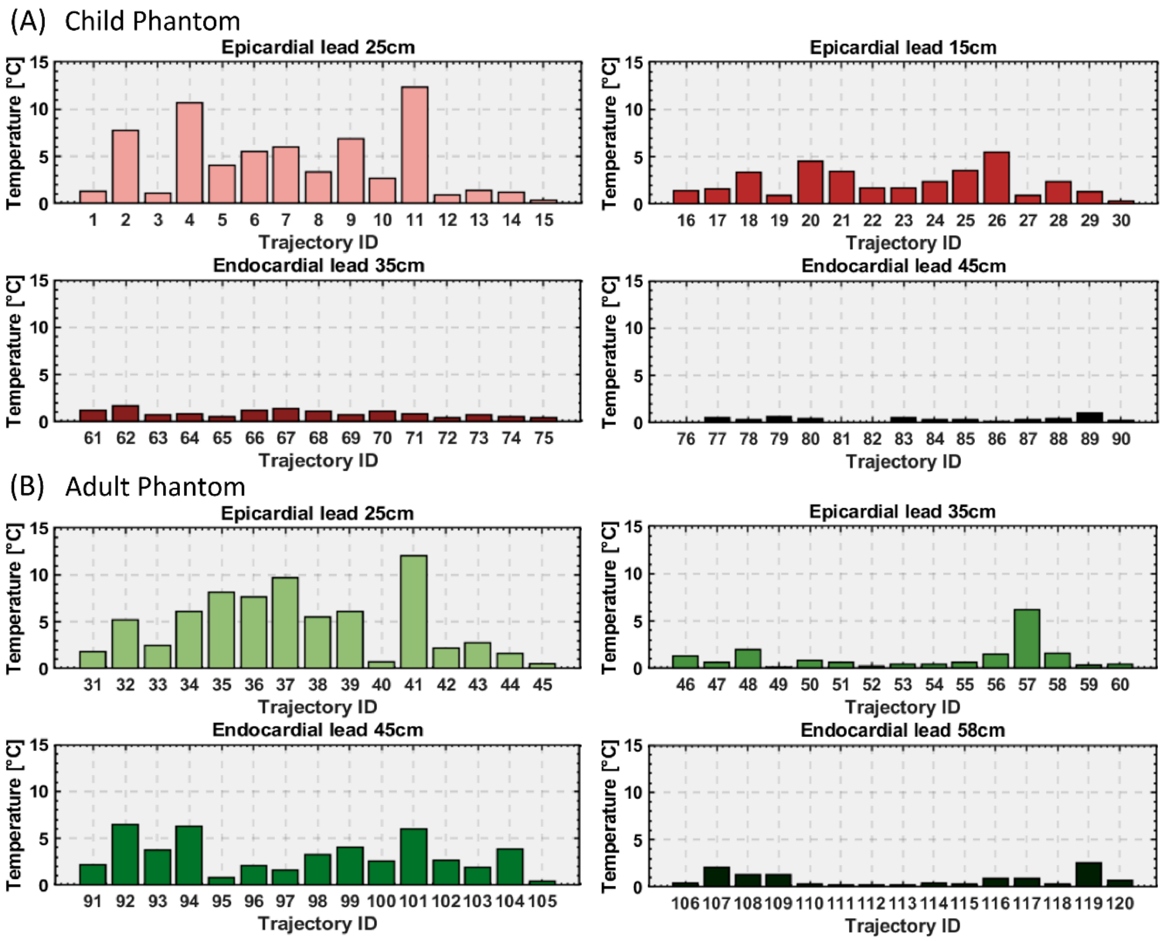

Figure S3: Temperature rise in the gel round tips of the leads with 120 distinct trajectories in pediatric and adult phantoms.

Supplementary Information Figure S4

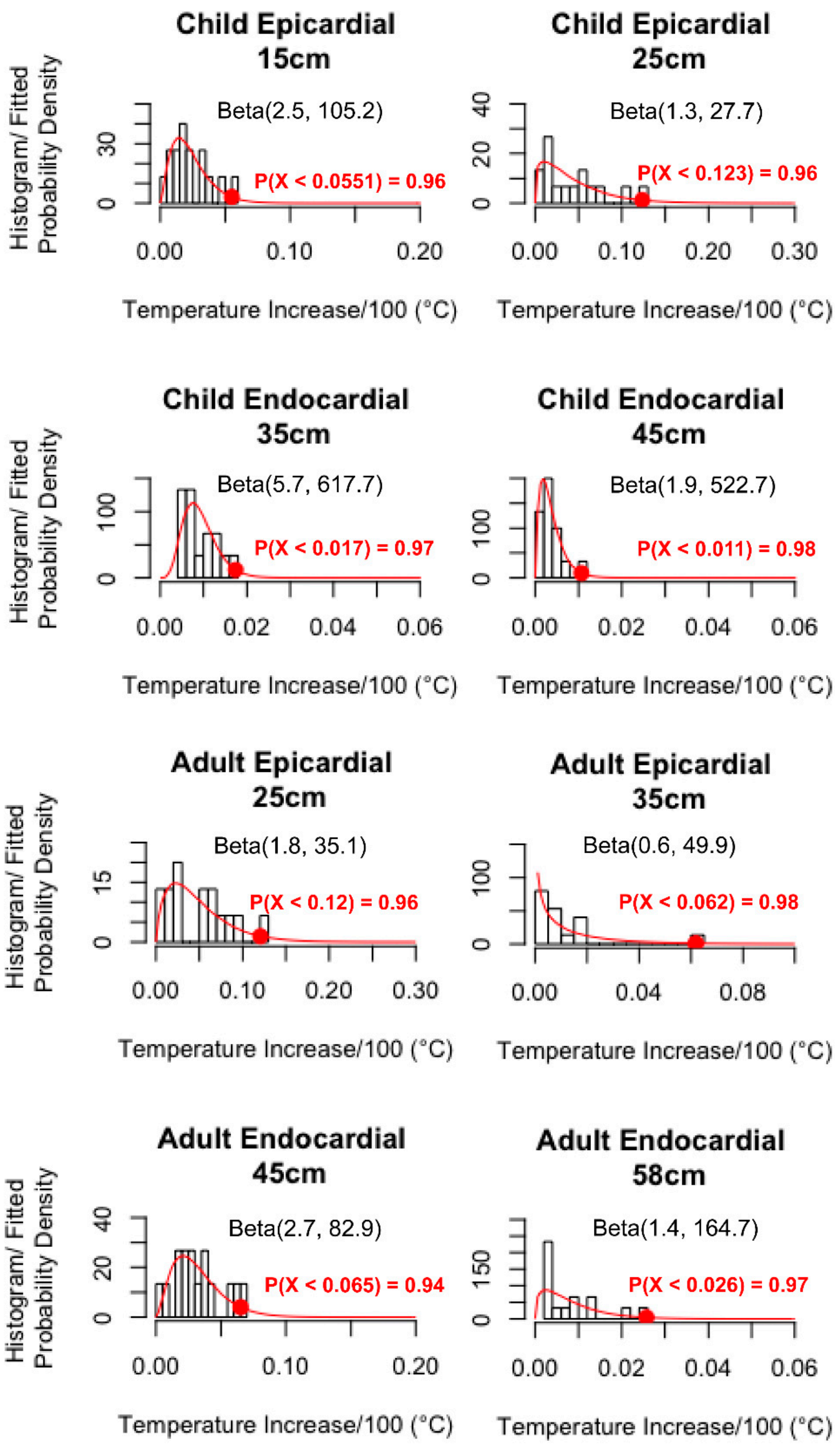

Figure S4: Histograms of scaled temperature rises. A beta probability density function was fitted to the data in each category. The probability of a measured temperature rise being below the highest heating determined during the experimental setup is shown for each scenario.
